# Pre-treatment Ongoing Cortical Oscillatory Activity Predicts Improvement of Tinnitus After Partial Peripheral Reafferentation With Hearing Aids

**DOI:** 10.3389/fnins.2020.00410

**Published:** 2020-05-07

**Authors:** Jae Joon Han, Dirk De Ridder, Sven Vanneste, Yu-Chen Chen, Ja-Won Koo, Jae-Jin Song

**Affiliations:** ^1^Department of Otorhinolaryngology-Head and Neck Surgery, Soonchunhyang University College of Medicine, Seoul Hospital, Seoul, South Korea; ^2^Unit of Neurosurgery, Department of Surgical Sciences, Dunedin School of Medicine, University of Otago, Dunedin, New Zealand; ^3^Lab for Clinical and Integrative Neuroscience, Trinity College of Neuroscience, Trinity College Dublin, Dublin, Ireland; ^4^Lab for Clinical and Integrative Neuroscience, School of Behavioral and Brain Sciences, The University of Texas at Dallas, Dallas, TX, United States; ^5^Department of Radiology, Nanjing First Hospital, Nanjing Medical University, Nanjing, China; ^6^Department of Otorhinolaryngology-Head and Neck Surgery, Seoul National University Bundang Hospital, Seoul, South Korea

**Keywords:** tinnitus, hearing aids, hearing loss, treatment, electroencephalography, neural plasticity

## Abstract

Although hearing aids (HAs) are sometimes efficacious in abating tinnitus, the precise mechanism underlying their effect is unclear and predictors of symptom improvement have not been determined. Here, we examined the correlation between the amount of tinnitus improvement and pre-HA quantitative electroencephalography (qEEG) findings to investigate cortical predictors of improvement after wearing HAs. QEEG data of thirty-three patients with debilitating tinnitus were retrospectively correlated with the percentage improvements in tinnitus handicap inventory and the numerical rating scale scores of tinnitus. Activation of brain areas involved in the default mode network (DMN; inferior parietal lobule, parahippocampus, and posterior cingulate cortex) were found to be a negative predictor of improvement in tinnitus-related distress after wearing HAs. In addition, higher pre-HA cortical power at the medial auditory processing system or higher functional connectivity of the lateral/medial auditory pathway to the DMN was found to serve as a positive prognostic indicator with regard to improvement of tinnitus-related distress. In addition, insufficient activity of the pre-treatment noise canceling system tended to be a negative predictor of tinnitus perception improvement after wearing HAs. The current study may serve as a milestone toward a pre-HAs prediction strategy for tinnitus improvements in subjects with hearing loss and severe tinnitus.

## Introduction

Tinnitus is an auditory symptom characterized by the perception of sound or noise in the absence of an external sound source. The overall prevalence of tinnitus in the general population is 10–15% (Henry et al., 2005; Bhatt et al., 2016) with about 7.2% of affected individuals reporting their symptom as a major problem and about half having discussed their symptom with a physician (Bhatt et al., 2016). Various treatment modalities, such as medication, sound therapy, cognitive behavioral therapy, tinnitus retraining therapy, cochlear implants, non-invasive brain stimulation, and hearing aids (HAs), have been suggested for the management of tinnitus (Langguth et al., 2013; Bae et al., 2020), yet no treatment exists that benefits all patients (Langguth et al., 2013).

Since Saltzman and Ersner first reported effectiveness of HAs for controlling tinnitus about 70 years ago (Saltzman and Ersner, 1947), many studies have supported the use of HAs as a treatment option for tinnitus (Kiessling, 1980; Brooks and Bulmer, 1981; Surr et al., 1985; Surr et al., 1999). Even though at a meta-analytic level there is no support for the use of HAs with or without maskers (Sereda et al., 2018), still approximately half of patients using HAs experience an improvement of tinnitus (Surr et al., 1999; Shekhawat et al., 2013). In addition, a recent review indicated that 17 of 18 original research studies showed positive evidence suggesting that using HAs for tinnitus management is beneficial (Shekhawat et al., 2013). Therefore, HAs have been recommended as a useful treatment option for tinnitus patients with hearing loss (Tunkel et al., 2014).

Several hypotheses have been proposed to explain the possible mechanism of tinnitus improvement associated with the use of HAs (Del Bo and Ambrosetti, 2007). For example, HAs have been suggested to be beneficial for tinnitus patients by helping the brain to distinguish between true sounds and tinnitus, decreasing annoyance by partial masking of tinnitus using augmented environmental sounds, and enhancing the ability to cope with tinnitus by reducing communication stress (Del Bo and Ambrosetti, 2007; Shekhawat et al., 2013). In addition, the neurophysiological rationale of HAs usage suggested that sound amplification by HAs may restore the activity of auditory neurons and cortical activity (Gabriel et al., 2006; Norena and Eggermont, 2006), and therefore may lead to neuroplastic changes of the auditory pathway and neural correlates related to the generation of tinnitus.

Previous researchers have suggested several mechanisms of tinnitus generation in subjects with reduced peripheral auditory input. That is, auditory deprivation may result in a compensation mechanism called “homeostatic plasticity,” a phenomenon affecting the neuronal activity of the auditory system at several levels along the ascending auditory pathway to compensate for the reduced peripheral input (Norena, 2011; Langguth et al., 2013). At the cortical level, peripheral deafferentation-induced changes such as reduced lateral inhibition in the deafferented cortical areas have been reported (Yang et al., 2011). Also, other researchers have suggested stochastic resonance serves to increase signals above the increased neuronal thresholds after peripheral hearing loss, thereby partly compensating for the hearing loss and possibly leading to the development of tinnitus (Krauss et al., 2016, 2018). Considering that all these models are based on the assumption that the ascending auditory system from the cochlea to the cerebral cortex attempts to maintain a neuronal network similar to the one before the peripheral auditory deprivation occurred, HA-assisted partial restoration of the cochlear input may abate tinnitus if these mechanisms are not totally irreversible. In other words, homeostatic plasticity along with reduced lateral inhibition or the stochastic resonance resulting in phantom auditory perception would become less active because less compensatory mechanisms are needed due to increased peripheral input assisted by HAs.

Hearing aid have long been proposed as one of the most useful treatment options for tinnitus, and most previous studies have reported positive effects of HAs on tinnitus. However, the mechanism underlying tinnitus improvement associated with HA usage remains unclear. In particular, despite previously suggested theories of tinnitus improvement by HAs implying the possible involvement of cortical neuroplastic changes, the neural substrates associated with the improvement of tinnitus by HAs have not been investigated. Based on previous studies of ours showing that pre-treatment cortical activities in the auditory cortex, posterior cingulate cortex, and the parahippocampus are important predictors of tinnitus improvement after hearing restoration by cochlear implantation (Song et al., 2013), and also showing that tinnitus in patients with hearing loss may be related to the parahippocampal mechanism (Vanneste and De Ridder, 2016), we surmised that similar areas might also be important in predicting tinnitus improvement associated with HA usage. The present study was performed to identify pre-treatment neural substrates associated with tinnitus improvement after using HAs in subjects with tinnitus. We also compared the cortical oscillatory activities of patients who showed marked improvement of tinnitus after wearing HAs with those who experienced slight improvement of tinnitus using source localization, complemented by connectivity analysis.

## Materials and Methods

### Participants

A total of 33 non-pulsatile subjective tinnitus subjects with hearing loss who had used HAs for more than 6 months were enrolled in this study. The mean age of the participants was 64.6 ± 14.6 years (range: 26–88 years), and 14 (42.4%) were male. During the initial visit, we obtained a structured medical history regarding the characteristics of tinnitus, such as the affected side, the psychoacoustic nature (pure-tone, narrow-band noise, or warble-tone), matched tinnitus frequency and loudness, and the duration of tinnitus perception. Out of the total of 33 subjects, 14 (42.4%) complained of unilateral tinnitus, while the remaining 19 had bilateral tinnitus. More than half of the participants (19 of 33, 57.6%) reported pure-tone tinnitus, while 11 (33.3%) had narrow-band noise, and one (3.0%) had warble-tone tinnitus. The most frequently matched frequency of tinnitus was 8 kHz (12 subjects, 36.4%), and the others showed variable tinnitus frequencies (4 kHz, five subjects; 3 kHz, one subject; 2 kHz, four subjects, 1 kHz, one subject; 500 Hz, four subjects; 250 Hz, two subjects). The mean duration of tinnitus perception was 8.4 ± 9.8 years (range: 3 months – 40 years). This retrospective study was approved by the Seoul National University Bundang Hospital Institutional Review Board (IRB) and the requirement for informed consent from the subjects was waived (IRB- B-1702/383-104) and the IRB waived the need for informed consent for this study. All methods were performed in accordance with the relevant guidelines and regulations.

The perceived tinnitus handicap was measured using the tinnitus handicap inventory (THI), a self-reported tinnitus handicap measure consisting of 25 items evaluating the extent of tinnitus-related distress (Newman et al., 1996). We evaluated tinnitus loudness (response to the question “How loud is your tinnitus?” on a scale from 0 to 10), tinnitus-related distress (response to the question “How bothered are you by your tinnitus?” on a scale from 0 to 10), and tinnitus perception (response to the question “What is the percentage of the daytime during which you are aware of the tinnitus?” from 0 to 100%) based on numerical rating scale (NRS) scores. The questionnaires were evaluated both before and after 6 months of using HAs. We excluded subjects with Ménière’s disease, pulsatile tinnitus, histories of drug or alcohol abuse, psychiatric/neurological disorders, and chronic headache; those using current psychotropic or central nervous system-active medications; those with a history of seizures; and those with any history of head injury resulting in loss of consciousness.

We used the median split method (Schlee et al., 2012; Song et al., 2013), a data-driven *post hoc* stratification approach, to compare the resting-state oscillatory brain activities between the subjects who showed marked improvement (MI group) and slight improvement (SI group) after tinnitus treatment with HAs. The MI and SI groups were assigned according to percentage improvement of NRS tinnitus distress scores. All subjects in the MI group showed >44.4% improvement of NRS tinnitus distress intensity, while all subjects in the SI group showed improvement <37.5%.

### Hearing Aids Fitting and Usage

At the initial visit, the hearing level of all participants was evaluated with pure tone audiometry (PTA) and speech audiometry, and all subjects were shown to have mild to severe hearing loss. The average hearing threshold (calculated by averaging the PTA thresholds at 500, 1000, 2000, and 4000 Hz) (Lee et al., 2017; Sunwoo et al., 2017; Song et al., 2018; Bae et al., 2019; Han et al., 2019; Huh et al., 2019; Lee et al., 2019b; Shim et al., 2019) of participants was 53.4±15.0 dB (range: 15.0 – 105.0) and the average speech discrimination score was 62.7 ± 25.7% (range: 12–100%). The selection of HAs was based on patients’ preferences and needs among all products available at our institute (Oticon, Resound, Siemens, Starkey, and Widex). The choice of HAs was also determined based on individual needs and choice from entry to advanced level. Therefore, the implemented technology and types of HAs were relatively variable. Fitting of HAs was performed by routine procedures according to each manufacturer’s guidelines and programmed via NOAH or NOAH-Link^®^ equipment using the algorithm provided by each manufacturer’s software. The presence of tinnitus and its matched frequency were not considered during the fitting process, and additional functions for tinnitus management, such as sound generation implemented in HAs, were not applied in this study so as to evaluate pure contribution of HAs to the improvement of tinnitus. HAs were fitted according to the individual’s configuration of hearing loss as well as to participant’s comfort and preference. The participants were counseled to increase the usage time of HAs gradually, up to full-time usage. Follow-up and re-fitting were performed 2 – 3 times a month until the patients were satisfied with the amplification of sound and alleviation of discomfort from HAs. After using HAs for over 6 months with proper fitting and full-time usage, aided threshold levels at frequencies of 250, 500, 1000, 2000, 3000, and 4000 Hz were evaluated. The average follow-up duration was 5.3 ± 0.9 months (range, 3 – 7 months). In addition, questionnaires on THI and NRS scores were obtained after 6 months of HA usage.

### EEG Recording

To identify the neural correlates related with tinnitus improvement after wearing HAs, we performed EEG recording before applying the HAs. Data acquisition and pre-processing procedures were performed according to the methods described previously (Song et al., 2017; Han et al., 2018; Vanneste et al., 2018; Lee et al., 2019a). Resting-state EEGs were recorded for about 5 min without any sound stimuli at 19 scalp sites using a tin electrode cap (ElectroCap, Eaton, OH, United States) connected to a Mitsar amplifier (Mitsar EEG-201; Mitsar, Saint Petersburg, Russia) in a fully lit room shielded from sound and stray electric fields. The participants sat upright on a comfortable chair during EEG recording. The EEG data were saved using WinEEG software (ver. 2.84.44; Mitsar)^1^. The impedances at all electrodes were maintained below 5 kΩ. Data were obtained at a sampling rate of 1024 Hz and then filtered using a high-pass filter with a cutoff of 0.15 Hz and a low-pass filter with a cutoff of 200 Hz. After initial data acquisition, the raw data were processed with resampling to 128 Hz and then with band-pass filtering using a fast Fourier transform filter and application of a Hanning window at 2 – 44 Hz. The data were then imported into Eureka! Software (Sherlin and Congedo, 2005). All episodic artifacts related to eye blinks, eye movements, teeth clenching, or body movements were inspected manually and removed from the EEG stream with Eureka! software. Further artifact removal was conducted by performing independent component analysis using ICoN software^2^ (Koprivova et al., 2011; White et al., 2012).

Caffeinated and alcoholic beverages were prohibited for 24 h prior to EEG recording to avoid caffeine-induced alpha and beta power decreases (Siepmann and Kirch, 2002) or alcohol-induced changes in the EEG stream (Volkow et al., 2000). Participants’ vigilance was monitored to prevent abnormal EEG signals, such as slowing of the alpha rhythm or enhancement of theta power (Moazami-Goudarzi et al., 2010). The participants included in the present study did not show drowsiness-related EEG changes.

### Source Localization Analysis

The localization of the intracerebral sources from the recorded EEG streams was performed using low-resolution brain electromagnetic tomography (LORETA)-KEY software^3^, a toolbox for the functional localization of standardized current densities based on electrophysiological and neuroanatomical constraints (Pascual-Marqui, 2002). Source localization was performed based on each of the following eight frequency bands: delta (2 – 3.5 Hz), theta (4 – 7.5 Hz), alpha1 (8 – 10 Hz), alpha2 (10 – 12 Hz), beta 1 (13 – 18 Hz), beta 2 (18.5 – 21 Hz), beta 3 (21.5 – 30 Hz), and gamma (30.5 – 44 Hz) bands (Kim et al., 2016; Song et al., 2017; Han et al., 2018; Vanneste et al., 2018). The electrical activity was computed as current density (μA/mm^2^) without assuming a predefined number of active sources. The LORETA-KEY software divides the Montreal Neurological Institute (MNI)-152 volume (Fuchs et al., 2002), including the hippocampus and anterior cingulate cortex, into 6239 voxels with dimensions of 5 mm × 5 mm × 5 mm. Scalp electrode coordinates on the MNI brain are derived from the international 5% system (Jurcak et al., 2007). Performing 5000 random permutations, correction for multiple testing (i.e., for tests performed for all electrodes and/or voxels, and for all time samples and/or different frequencies) was carried out and, thus, no further correction for multiple comparison was needed. LORETA-KEY assumes related orientations and strengths of neighboring neuronal sources and, thus, the inverse problem, which is derived from source reconstruction from electric neuronal activity based on extracranial measurements, is solved by the algorithm. Anatomical labeling of significant clusters was performed automatically by an embedded toolbox within LORETA-KEY, and these labels were reconfirmed by matching the centroids of significant clusters with the Talairach and Tournoux atlas (Talairach and Tornoux, 1988).

### Functional Connectivity Analysis

Phase synchronization and the extent of coherence between the MI group and SI group corresponding to different regions of interest (ROIs) were calculated to evaluate the difference of functional connectivity between two groups using the LORETA-KEY connectivity toolbox. This toolbox defines measures of linear and non-linear dependence (i.e., coherence and phase synchronization) between multivariate time series; the measures are expressed as the sums of lagged/instantaneous dependencies. For functional connectivity analysis, a total of 16 ROIs defined by the Brodmann area (BA), known to be related to tinnitus according to the literature, were selected as possible nodes: the bilateral primary auditory cortices (A1, BAs 41 and 42) (Rolls, 2004; Kringelbach, 2005; Smits et al., 2007), the bilateral parahippocampus (PHC, BA 27) (Landgrebe et al., 2009), the bilateral dorsal/pregenual/subgenual anterior cingulate cortices (dACC, BA 24; pgACC, BA 32; sgACC, BA 25) (Damasio, 1996; Vanneste et al., 2010; De Ridder et al., 2011b), the bilateral orbitofrontal cortices (OFC, BA 10) (Song et al., 2015), and the bilateral inferior parietal lobule (IPL, BA 40) (Houdayer et al., 2015; Chen et al., 2018).

### Region of Interest (ROI) Analysis

For ROIs that showed a significant correlation with the percentage improvements in NRS tinnitus-related distress or perception, log-transformed electric current density was averaged. Each ROI was defined by a single voxel that was closest to the center of the area where a significant difference was found in the source localization analysis. Then, the correlation between the log-transformed electric current density in each ROI and the percentage improvements in NRS tinnitus-related distress or perception was evaluated by calculating the Pearson correlation coefficient.

### Statistical Analysis

To identify the resting-state cortical oscillatory activities related to tinnitus improvement, the LORETA-KEY images were analyzed by the statistical non-parametric mapping (SnPM) method for each contrast using LORETA-KEY’s built-in voxel-wise randomization tests (5000 permutations). In this way, we correlated pre-HAs source-localized activities among whole-brain areas and a connectivity of 16 ROIs with the percentage improvement in the THI score, NRS loudness, NRS distress, and NRS perception. Also, to test the reliability of our results, we have split the enrolled subjects into 2 groups according to the order of enrollment (an odd-numbered subject group and an even-numbered subject group) and performed the same analyses to see if the correlations are maintained in these two subgroups. Moreover, we employed a *t* statistic between MI and SI groups with a threshold of *P* < 0.05. Correction for multiple comparisons in SnPM using random permutations has been shown to yield similar results to those obtained from a statistical parametric mapping approach using a general linear model with multiple comparison corrections (Nichols and Holmes, 2002). For lagged linear connectivity differences, we compared the differences between the MI and SI groups for each contrast using the paired *t* statistic with a threshold of *P* < 0.05. We also corrected for multiple comparisons using LORETA-KEY’s built-in voxel-wise randomization tests on all of the voxels included in the 16 ROIs for connectivity analysis (5000 permutations). All other descriptive statistical analyses were performed using SPSS software (version 20.0, SPSS, Inc., Chicago, IL, United States). In all analyses, *P* < 0.05 was taken to indicate statistical significance.

## Results

### Functional Gain and Tinnitus Improvement

For 33 tinnitus participants included, the average aided hearing thresholds with HAs were significantly better than unaided air conduction thresholds at all frequencies [250 Hz, 27.0 ± 10.6 vs. 33.9 ± 19.0; 500 Hz, 31.2 ± 11.9 vs. 38.6 ± 17.5; 1 kHz (32.0 ± 12.3 vs. 50.6 ± 18.7; 2 kHz, 37.7 ± 13.1 vs. 56.1 ± 19.2; 3 kHz, 44.7 ± 13.9 vs. 63.3 ± 18.4; 4 kHz, 50.2 ± 13.7 vs. 68.3 ± 19.4; *P* < 0.01 for all frequencies)] (Supplementary Figure S1). The average functional gains of HAs were 7.0 ± 14.7 dB at 250 Hz, 7.4 ± 12.6 dB at 500 Hz, 18.6 ± 12.2 dB at 1 kHz, 18.3 ± 14.1 dB at 2 kHz, 18.6 ± 12.8 dB at 3 kHz, and 18.2 ± 12.7 dB at 4 kHz.

All questionnaire scores pertaining to tinnitus severity improved significantly after using HAs (Table 1). The mean THI scores after using HAs were significantly lower than pre-HA THI scores (27.9 ± 18.0 vs. 56.8 ± 24.0, respectively, *P* < 0.01). In addition, NRS tinnitus loudness (4.8 ± 2.1 vs. 7.0 ± 2.1, respectively, *P* < 0.01), tinnitus distress (4.3 ± 2.6 vs. 6.7 ± 3.0, respectively, *P* < 0.01), and tinnitus perception (59.7 ± 36.4 vs. 79.1 ± 27.5, respectively, *P* < 0.01) improved significantly after 6 months of wearing HAs. To evaluate the relationship between the degree of tinnitus improvement and the functional gain with HAs, we performed simple correlation analysis between the functional gains and the tinnitus questionnaire scores. The percentage improvements in the THI scores were not significantly correlated with the average functional gain with use of HAs (*r* = 0.02, *P* = 0.92). The percentage changes of NRS loudness (*r* = −0.13, *P* = 0.48), distress (*r* = 0.049, *P* = 0.78), and perception (*r* = −0.01, *P* = 0.94) were also not significantly correlated with the functional gain in use of HAs.

**TABLE 1:** Changes in tinnitus handicap inventory and numeric rating scale scores 6 months after using hearing aids.

	Pre-HAs	Post-HAs	*P*-value
THI score	56.824.0	27.918.0	<0.01
NRS loudness	7.02.1	4.82.1	<0.01
NRS distress	6.73.0	4.32.6	<0.01
NRS perception (%)	79.127.5	59.736.4	<0.01

*THI, tinnitus handicap inventory; NRS, numeric rating scale; HAs, hearing aids.*

### Source-Localized Correlation Analysis Between Pre-HAs qEEG and Post-HAs Tinnitus Improvement

**FIGURE 1:**
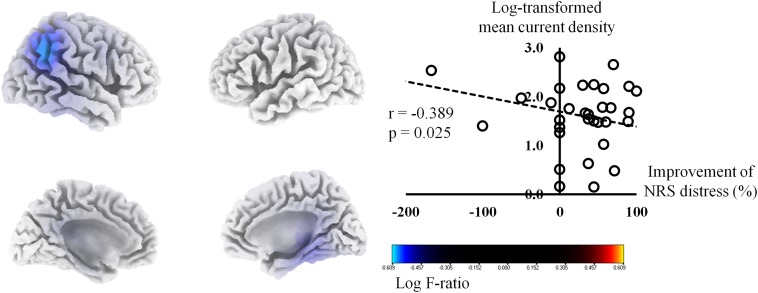
Source-localized correlation analysis between the percentage improvements in the numerical rating scale (NRS) of tinnitus distress and the resting-state quantitative electroencephalography data before wearing hearing aids (HAs). The percentage improvements in NRS tinnitus-related distress correlated negatively with the pre-HA source-localized activities at the right inferior parietal lobule, right parahippocampus, and right posterior cingulate cortex for the gamma frequency band.

The percentage improvements in NRS tinnitus-related distress were negatively correlated with the pre-HA source-localized activities at the right IPL (BA 40), right PHC (BA 27), and right posterior cingulate cortex (PCC, BA 23) for the gamma frequency band (*P* < 0.01) (Figure 1). The correlation analysis between the log-transformed mean current density for the gamma frequency band at BA 40 and the percentage improvements in NRS tinnitus-related distress showed a significant negative correlation (*r* = −0.39, *P* = 0.03). In subgroup analysis, we have confirmed negative correlations between the percentage improvements in NRS tinnitus-related distress and the right IPL, PHC, and PCC in each subgroup (Supplementary Figure S2), in accordance with the results for all subjects (Figure 1). For the other seven frequency bands, there were no significant correlations between the percentage improvements of NRS distress and the pre-HA source-localized activities.

**FIGURE 2:**
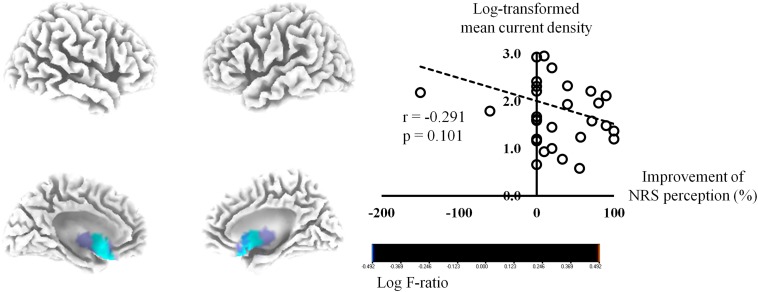
Source-localized correlation analysis between the percentage improvements in the numerical rating scale (NRS) of tinnitus perception and the resting-state quantitative electroencephalography data before wearing hearing aids (HAs). The activities of the bilateral subgenual anterior cingulate cortex exhibited negative correlations of marginal significance with the percentage improvements in NRS tinnitus perception for the beta 3 frequency band (*P* = 0.08).

The activities of the bilateral sgACC (BA 25) exhibited negative correlations of marginal significance with the percentage improvements in NRS tinnitus perception for the beta 3 frequency band (*P* = 0.08) (Figure 2). The log-transformed mean current density for the beta 3 frequency band at the right BA 25 showed a tendency of negative correlation with the percentage improvements in NRS tinnitus-related perception (*P* = 0.10). In subgroup analysis, the tendency of negative correlation between the percentage improvements in NRS tinnitus-related perception and the bilateral sgACC for beta 3 frequency band was also confirmed in each subgroup (Supplementary Figure S3), in accordance with the results for all subjects (Figure 2). No significant correlations were evident between the percentage improvements of NRS perception and the pre-HA source-localized activities for the other seven frequency bands.

Meanwhile, the percentage improvement of NRS tinnitus loudness did not show significant correlations with pre-HA source-localized activities at any of the eight frequency bands examined.

**FIGURE 3:**
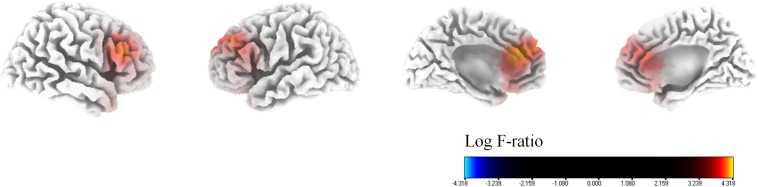
Median-split source-localized comparison between the marked improvement (MI) group and the slight improvement (SI) group with regard to tinnitus-related distress. The MI group showed significantly higher cortical powers in the bilateral dorsolateral prefrontal cortices, bilateral dorsal anterior cingulate cortices, and bilateral subgenual anterior cingulate cortices for the alpha 1 frequency band in comparison with the SI group.

With regard to the percentage improvement of NRS tinnitus distress, in comparison to the SI group, the MI group showed significantly higher cortical powers in the bilateral dorsolateral prefrontal cortices (DLPFC, BA 9), bilateral dACC (BA 24), and bilateral sgACC (BA 32) for the alpha 1 frequency band (*P* < 0.01) (Figure 3). There were no significant differences between the two groups for the other seven frequency bands (delta, theta, alpha 2, beta 1, 2, and 3).

### Connectivity Analyses

**FIGURE 4:**
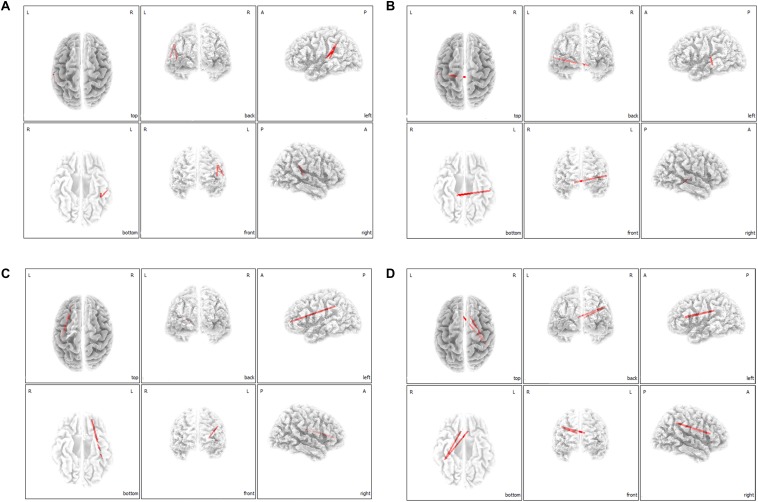
Median-split lagged linear functional connectivity analysis with regard to tinnitus-related distress. The marked improvement (MI) group showed significantly increased functional connectivities in comparison with the slight improvement (SI) group between the left primary auditory cortices and left inferior parietal lobule for the delta frequency band **(A)**, between the left primary auditory cortex and right parahippocampus for the theta frequency band **(B)**, between the left orbitofrontal cortex and left inferior parietal lobule for the alpha 1 frequency band **(C)**, and between the bilateral dorsal anterior cingulate cortex and right inferior parietal lobule for the alpha 2 frequency band **(D)**.

On correlation analysis between lagged linear functional connectivity of the 16 ROIs and questionnaire scores (THI and NRS scores), no significant correlations were found. However, on lagged linear functional connectivity analysis with regard to tinnitus-related distress, the MI group showed significantly increased functional connectivities in comparison to the SI group between the left A1 and left IPL for the delta frequency band (Figure 4A), between the left A1 and right PHC for the theta frequency band (Figure 4B), between the left OFC and left IPL for the alpha 1 frequency band (Figure 4C), and between the bilateral dACC and right IPL for the alpha 2 frequency band (Figure 4D) (*P* < 0.05). For the other four frequency bands (beta 1, 2, 3, and gamma), no significant differences were found between the MI and SI groups with regard to functional connectivity among the 16 ROIs.

## Discussion

In the current study, we sought to evaluate the treatment effect of HAs in subjects with debilitating tinnitus and to gain a better understanding of the underlying neural predictors of a beneficial effect related to HAs. We observed significant tinnitus improvement 6 months after wearing HAs and found several positive- and negative pre-treatment cortical indicators that may be important for predicting the amount of improvement with regard to tinnitus-related distress and tinnitus perception during the daytime. In addition to previous observational studies showing improvement of tinnitus after wearing HAs in subjects with tinnitus, the current study is the first to demonstrate treatment outcome may be predictable based on the status of the brain before HAs are applied.

### Activation of the Default Mode Network as a Negative Predictor of Tinnitus-Related Distress Improvements Even After Partial Peripheral Reafferentation by HAs

As described above, the percentage improvements in NRS tinnitus-related distress showed statistically significant negative correlations with the pre-HAs source-localized activities at the right IPL, right PHC, and right PCC for the gamma frequency band (Figure 1). These three areas have been regarded as the components of the brain’s default mode network (DMN) (Raichle et al., 2001; Raichle and Snyder, 2007; Raichle, 2015), a specific group of brain regions activated when people are occupied with internally focused tasks.

Previous studies have suggested that tinnitus generators may become integrated in the DMN in subjects with tinnitus (De Ridder et al., 2011a; Vanneste and De Ridder, 2012). In this regard, an abnormally increased activity in tinnitus patients with hearing loss may be an ominous sign with regard to symptom improvement after wearing HAs. That is, even after partial reafferentation by HAs, tinnitus-related distress may not be successfully improved because tinnitus itself may already have become a norm due to activation of the components of the DMN. This is also consistent with a previous report showing relatively unsatisfactory improvement of tinnitus after cochlear implantation in single-sided deafness subjects who showed relatively high preoperative cortical power at the components of the DMN (Song et al., 2013).

### Tinnitus Subjects With Higher Medial Auditory Processing System Activity May Respond More to HA-Assisted Increased Auditory Input

On median split analysis with regard to tinnitus-related distress, the MI group showed significantly higher cortical powers in the bilateral DLPFC, bilateral dACC, and bilateral sgACC for the alpha 1 frequency band in comparison with the SI group (Figure 3). Analogous to the medial pain system (Price, 2000; Kulkarni et al., 2005), a medial auditory processing system in charge of affective components of sound processing has recently been proposed (Leaver et al., 2011; De Ridder et al., 2014). As the DLPFC, dACC, and sgACC are components of the medial auditory processing system, we posit that tinnitus subjects with higher medial auditory processing system activity may respond better to HA-assisted increased auditory input. That is, partial peripheral reafferentation by HAs may abate the affective component of tinnitus to a greater extent in subjects with abnormally increased activity in the affective sound processing system.

This is also consistent with a previous electrical stimulation study indicating activation of the DLPFC and the dACC in patients with limb and back pain undergoing burst stimulation of the spinal cord (De Ridder et al., 2013). As the DLPFC and the dACC are also components of the medial pain pathway (De Ridder et al., 2013), the results of the present study showing higher cortical powers in the DLPFC and dACC in the MI group reconfirm previous reports suggesting analogies between chronic pain and tinnitus (Tonndorf, 1987; De Ridder et al., 2011a; Rauschecker et al., 2015). That is, in addition to previously suggested analogies, such as central phantom perception after peripheral injury or hypersensitivity to sensory stimulation, both chronic pain and tinnitus may respond well to peripheral stimulation when the medial pain or auditory pathway is activated.

### Functional Connection of the Lateral/Medial Auditory Pathway to the IPL and PHC May Ameliorate the Negative Influence of the DMN With Regard to the Improvement of Tinnitus-Related Distress

Functional connectivity contrast analysis between the MI and SI groups revealed significantly increased functional connectivities between the left A1 and the left IPL in the MI group compared to those in the SI group for the delta frequency band (Figure 4A), and between the left A1 and right PHC for the theta frequency band (Figure 4B). Together with the findings of source-localized correlation analysis showing higher pre-HA cortical powers at the IPL and the PHC as negative prognostic factors with regard to improvement of tinnitus-related distress after wearing HAs, the median split connectivity analysis results may indicate that tinnitus distress would be abated more if the IPL and PHC, the components of DMN, are functionally connected to A1. That is, if the lateral auditory pathway is activated by HA-assisted partial peripheral reafferentation and if A1 is functionally connected to the IPL or the PHC, this may offset the negative influence of the IPL and the PHC with regard to improvement of tinnitus-related distress after wearing HAs. In this regard, increased functional connectivity between the bilateral dACCs and right IPL for the alpha 2 frequency band in the MI group, as compared to the SI group, could also be interpreted in the same way. That is, if a tinnitus subject’s functional connection between the medial auditory pathway and the DMN is relatively increased before wearing HAs, there is a relatively greater chance of tinnitus-related distress improvement after wearing HAs because abnormally increased activity in the DMN may be mitigated by increased activity of the medial auditory pathway after wearing HAs. This is in agreement with direct modulation of the dACC and auditory cortex by implants. If a patient has increased functional connectivity between the auditory cortex and the parahippocampus, the patient is more likely to respond to the implant on the auditory cortex (De Ridder and Vanneste, 2014). Similarly, if a patient has increased functional connectivity from the dACC, an implant is more likely to be effective in improving tinnitus (De Ridder et al., 2016).

### Pre-treatment Noise-Canceling System Activity May Determine the Improvement of Tinnitus Perception After Wearing Hearing Aids

The percentage improvements in NRS tinnitus-related perception showed a trend toward a negative correlation (*P* = 0.08) with the pre-HA source-localized activities at the bilateral sgACC (Figure 2). Although not statistically significant, these results were consistent with our previous report positing the sgACC as one of the core components of the descending noise-canceling mechanism that counteracts peripheral auditory deafferentation-based phantom sound (i.e., tinnitus) generation (Lee et al., 2019a). That is, our results suggest that if the pre-treatment cortical oscillatory power of the noise canceling system consisting of the rostral ACC as well as the sgACC is not sufficient, the perception of tinnitus may not be improved even after partial restoration of the auditory input by HAs. This is another analogy with chronic pain. That is, when performing spinal cord stimulation, the amount of improvement depends on the activation of the pgACC (Moens et al., 2012), which is part of the descending pain inhibitory pathway (Fields, 2004; Kong et al., 2010), the somatosensory analog of the noise canceling system.

The sgACC itself extends into the ventromedial prefrontal cortex (vmPFC), which was suggested in previous functional MRI studies to form part of the core of tinnitus perception (Leaver et al., 2011, 2012). Other groups have proposed that the vmPFC and the nucleus accumbens are part of a central “gatekeeping” system that evaluates the relevance of sensory stimuli and modulates information flow via descending pathways (Rauschecker et al., 2015). In this regard, it was suggested that tinnitus occurs when the function of the vmPFC is compromised. Considering the anatomical proximity between the sgACC and vmPFC, we conjecture that a dysfunctional sgACC (or vmPFC) may be responsible for the failure of descending noise canceling and, thus, induction of tinnitus perception. Judging from the results of the present study showing a trend-level negative correlation between pre-HA sgACC activities and post-HA improvements in tinnitus perception (Figure 2), we surmised that partial reafferentation by HAs may not be effective for tinnitus subjects with dysfunctional sgACCs with regard to the reduction of tinnitus perception.

### Strengths and Limitations of This Study

To our knowledge, this is the first study to show the possibility of treatment outcome prediction with regard to the amount of tinnitus improvement using pre-HA cortical oscillatory features.

However, several limitations of the present study should be noted. First, as we evaluated the treatment outcomes of HAs 6 months after the first fitting, the long-term effects of HAs with regard to improvement of tinnitus should be re-evaluated in a follow-up study. That is, future studies correlating pre-treatment rs-qEEG data with long-term outcomes should be conducted to determine the cortical predictors of the final tinnitus treatment outcome after wearing HAs. Second, information on the post-HA status of the included subjects was limited because only questionnaires, such as THI and NRS scales, were used for outcome evaluation of tinnitus improvement. In this regard, future follow-up studies exploring post-HA qEEG changes to determine objective cortical oscillatory activity changes after partial peripheral reafferentation by HAs, and to correlate changes in cortical oscillatory activities with changes in subjective tinnitus and tinnitus-related distress, should be performed. Third, we enrolled tinnitus patients with severe distress (i.e., mean THI score of 56.8, NRS loudness of 7.0, and NRS distress of 6.7). That is, only a few subjects had mild distress in this retrospective cohort. To explore HA-related tinnitus improvement and relevant cortical activity changes, and to minimize the effects of improved distress, further studies are required to explore subjective symptom changes and objective oscillatory power changes in subjects with tinnitus, with minimal distress caused by their symptoms. Fourth, dividing the subjects into groups with- and without meaningful THI improvements, and comparing pre-HAs qEEGs to see the pre-treatment differences between good- and poor responders to HAs would give us further valuable information on the clinical setting. However, there were only 3 of 33 subjects who showed clinically an “improvement,” defined as a decrease of more than 20% in the THI score (Khedr et al., 2008), and thus the current study group precluded the possibility of the group comparison between good- and poor responders. Future follow-up studies in a larger number of subjects are warranted to discover pre-HAs predictors of clinically significant improvements in tinnitus after wearing HAs.

## Conclusion

Taken together, we evaluated the effects of HAs in subjects with severe tinnitus, with reference to pre-treatment cortical oscillatory patterns measured by rs-qEEG. This study underscored activation of the DMN as a negative predictor of tinnitus-related distress improvements even after partial peripheral reafferentation by HAs. In addition, we found that higher pre-HA cortical oscillatory power at the medial auditory processing system or functional connection of the lateral/medial auditory pathway to the DMN may act as positive prognostic indicators with regard to improvement of tinnitus-related distress. Finally, we observed a tendency toward insufficient activity of the pre-treatment noise canceling system as a negative predictor of tinnitus perception improvement after wearing HAs. The present study may facilitate the development of a pre-HA prediction strategy for tinnitus improvements in subjects with hearing loss and severe tinnitus.

## Data Availability Statement

The datasets generated for this study are available on request to the corresponding author.

## Ethics Statement

The studies involving human participants were reviewed and approved by Seoul National University Bundang Hospital Institutional Review Board. Written informed consent for participation was not required for this study in accordance with the national legislation and the institutional requirements.

## Author Contributions

J-JS led the analysis and interpretation of the result, and drafted the first manuscript. JH and J-JS conceived the investigation, revised the manuscript for important intellectual content. JH, DR, SV, Y-CC, J-WK, and J-JS contributed to all aspects of the investigation, including methodological design, data collection and analysis, interpretation of the results, and revision of the manuscript for important intellectual content. All authors approved the final version of the manuscript and agreed to be accountable for all aspects of the work.

## Conflict of Interest

The authors declare that the research was conducted in the absence of any commercial or financial relationships that could be construed as a potential conflict of interest.

## Footnotes

1 http://www.mitsar-medical.com

2 http://sites.google.com/site/marcocongedo/software/nica

3 http://www.uzh.ch/keyinst/NewLORETA/Software/Software.htm
