# Supplementary material for: Pre-treatment Ongoing Cortical Oscillatory Activity Predicts Improvement of Tinnitus After Partial Peripheral Reafferentation With Hearing Aids
Source: Front Neurosci. 2020 May 7;14:410. doi: 10.3389/fnins.2020.00410 (PMC7221249; doi:10.3389/fnins.2020.00410)
Supplement: Supplementary file 1 [file Data_Sheet_1.docx]

**Supplemental information**

**Title: Pre-treatment ongoing cortical oscillatory activity predicts improvement of tinnitus after partial peripheral reafferentation with hearing aids**

Authors:

Jae Joon Han, MD, Dirk De Ridder, MD, PhD, Sven Vanneste, PhD, Yu-Chen Chen, Ja-Won Koo, MD, PhD, Jae-Jin Song, MD, PhD*

^*^corresponding. [jjsong96@gmail.com](mailto:jjsong96@gmail.com)


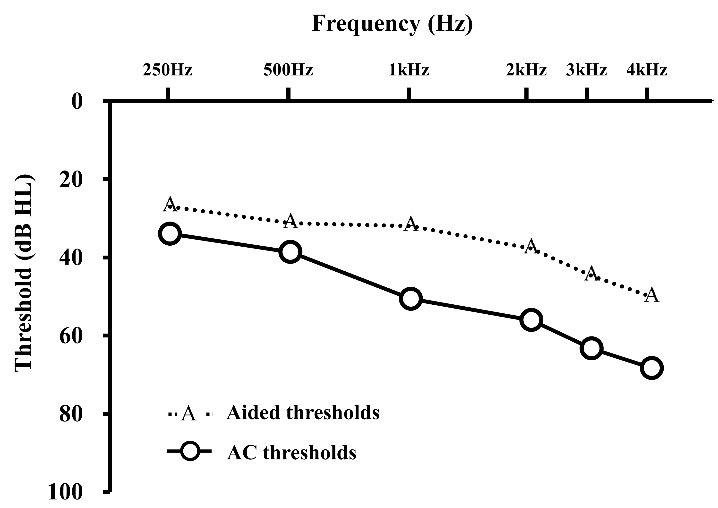


**Supplementary Figure S1.** Unaided air conduction thresholds and aided hearing thresholds with hearing aids. The hearing thresholds of the participants are successfully improved after wearing hearing aids, and the functional gain are about 7 dB at low frequencies (250, 500 Hz) and 18 dB at mid and high frequencies (1, 2, 3, 4 kHz).


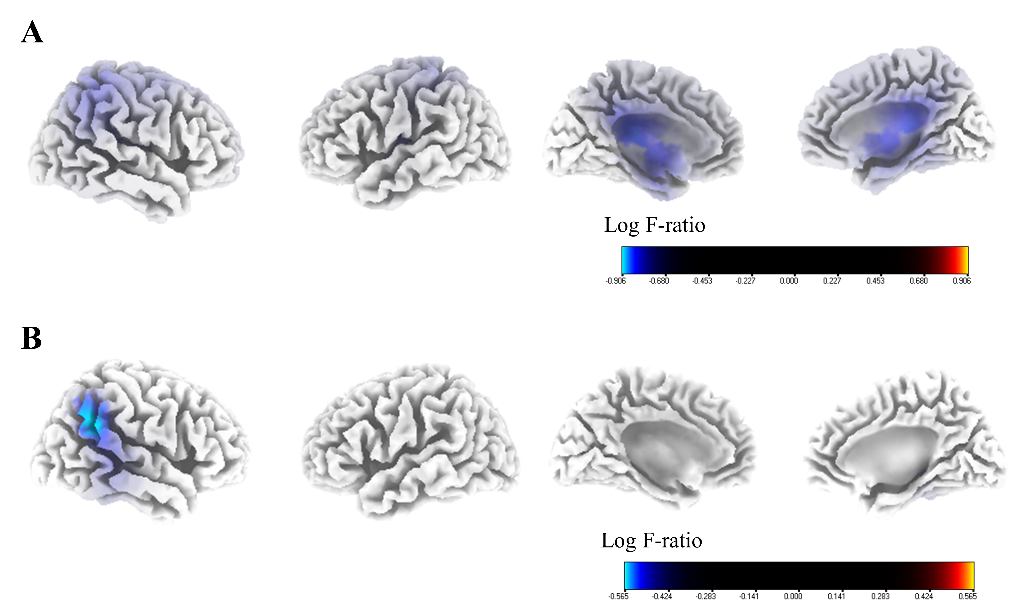


**Supplementary Figure S2. Source-localized correlation analysis between the percentage improvements in the numerical rating scale (NRS) of tinnitus-related distress and the resting-state quantitative electroencephalography data before wearing hearing aids (HAs) in subgroup analysis.** In both subgroups (A and B) divided by the odd- or even-number of enrollment order, the percentage improvements in NRS tinnitus-related distress correlated negatively with the pre-HA source-localized activities at the right inferior parietal lobule, right parahippocampus, and right posterior cingulate cortex for the gamma frequency band.


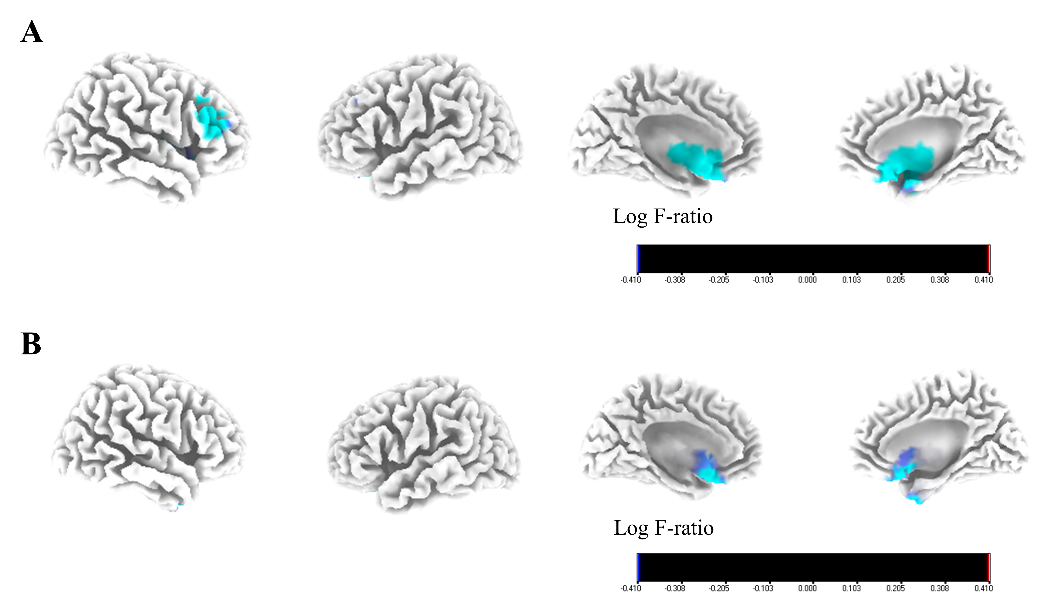


**Supplementary Figure S3. Source-localized correlation analysis between the percentage improvements in the numerical rating scale (NRS) of tinnitus perception and the resting-state quantitative electroencephalography data before wearing hearing aids (HAs) in subgroup analysis.** In both subgroups (A and B) divided by the odd- or even-number of enrollment order, the activities of the bilateral subgenual anterior cingulate cortex exhibited marginally significant negative correlations with the percentage improvements in NRS tinnitus perception for the beta 3 frequency band.
